# Ischemic Postconditioning-Mediated DJ-1 Activation Mitigate Intestinal Mucosa Injury Induced by Myocardial Ischemia Reperfusion in Rats Through Keap1/Nrf2 Pathway

**DOI:** 10.3389/fmolb.2021.655619

**Published:** 2021-04-30

**Authors:** Rong Chen, Wei Li, Zhen Qiu, Qin Zhou, Yuan Zhang, Wen-yuan Li, Ke Ding, Qing-tao Meng, Zhong-yuan Xia

**Affiliations:** Department of Anesthesiology, Renmin Hospital of Wuhan University, Wuhan, China

**Keywords:** intestinal barrier injury, ischemic postconditioning, DJ-1, myocardial ischemia reperfusion, nuclear factor (erythroid-derived 2)-like 2

## Abstract

Intestinal mucosal barrier dysfunction induced by myocardial ischemia reperfusion (IR) injury often leads to adverse cardiovascular outcomes after myocardial infarction. Early detection and prevention of remote intestinal injury following myocardial IR may help to estimate and improve prognosis after acute myocardial infarction (AMI). This study investigated the protective effect of myocardial ischemic postconditioning (IPo) on intestinal barrier injury induced by myocardial IR and the underlying cellular signaling mechanisms with a focus on the DJ-1. Adult SD rats were subjected to unilateral myocardial IR with or without ischemic postconditioning. After 30 min of ischemia and 120 min of reperfusion, heart tissue, intestine, and blood were collected for subsequent examination. The outcome measures were (i) intestinal histopathology, (ii) intestinal barrier function and inflammatory responses, (iii) apoptosis and oxidative stress, and (iv) cellular signaling changes. IPo significantly attenuated intestinal injury induced by myocardial IR. Furthermore, IPo significantly increased DJ-1, nuclear Nrf2, NQO1, and HO-1 expression in the intestine and inhibited IR-induced apoptosis and oxidative stress. The protective effect of IPo was abolished by the knockdown of DJ-1. Conversely, the overexpression of DJ-1 provided a protective effect similar to that of IPo. Our data indicate that IPo protects the intestine against myocardial IR, which is likely mediated by the upregulation of DJ-1/Nrf2 pathway.

## Introduction

Cardiovascular disease (CVD) [e.g., myocardial infarction (MI) and stroke] is the leading cause of heart failure (HF) (Pagliaro et al., 2020) and cardiac death worldwide. Acute myocardial infarction (AMI) is related to a decrease in cardiac output and mean aortic pressure, which induces episode of acute decompensated HF and then reduces the visceral blood volume, especially in the intestine (Wang et al., 1996). The splanchnic circulation accepts approximately 25% of cardiac outputs in the resting state, and the mucosal layer receives more than two-thirds of the bowel wall blood flow because of the high metabolic demand. This special structure makes it extremely sensitive to the reduction in cardiac output (Kalder et al., 2015). Although therapeutic approaches involving reperfusion by percutaneous coronary intervention (PCI) and thrombolysis have reduced the acute mortality rates of AMI, increasing evidence has showed that the incidence of other organ damage events post-AMI still predicts an increased mortality (Heusch and Gersh, 2017). The process of recovering blood flow to the ischemic myocardium can induce myocardial ischemia reperfusion (IR) injury (Neri et al., 2017). Intestinal ischemia caused by AMI combined with oxidative stress and inflammation caused by myocardial IR injury result in intestinal mucosal barrier dysfunction, which is eventually causing the dysbiosis and translocation of bacteria, amplifying the inflammatory response to promote the progression of CVD (Brown and Hazen, 2015), and damaging other remote organs, which will lead to a poor prognosis for patients. Therefore, early detection and prevention of remote intestinal injury following myocardial IR may help to predict and improve the prognosis of these patients.

Ischemic postconditioning (IPo) refers to brief transient episodes of ischemia and reperfusion; a maneuver performed immediately at the onset of reperfusion has been shown to be effective in reducing IR injury in many organs [e.g., the heart (Hao et al., 2017), kidney (Liu et al., 2020), lung (Gao et al., 2017), and intestine (Meng et al., 2016, p. 2)]. IPo could significantly reduce the myocardial infarct size and decrease the level of biomarkers (creatine kinase–muscle brain, or troponin) (Xue et al., 2016; Wang et al., 2019). This endogenous myocardial protective-adaptive intervention suppresses spontaneous Ca^2+^ oscillations and delays the restoration of cytosolic pH, and these events lead to a reduction in the mitochondrial reactive oxygen species (ROS) generation (Shahzad et al., 2013). However, whether the myocardial IPo can effectively protect the intestine from myocardial IR and the possible protective mechanisms involved are not fully understood.

DJ-1 [also known as Parkinson’s disease (autosomal recessive, early onset) 7 (PARK7)] was originally found to be linked to an early-onset autosomal recessive form of Parkinson’s disease (Bonifati et al., 2003). In recent years, it has been confirmed that DJ-1 can function as a ubiquitous cytoprotective protein that plays a vital role in multiple cellular processes, such as oxidative stress, protein quality control, antiapoptotic signaling, and transcriptional regulation (Wang et al., 2012; Amatullah et al., 2017). Studies have shown that the DJ-1 protein can provide cytoprotection against myocardial IR injury (Shimizu et al., 2016). DJ-1 deficiency increases the susceptibility to cell death from myocardial acute IR, as evidenced by a larger MI size (Dongworth et al., 2014). As a key factor in the oxidative stress response of cells, NF-E2-related factor 2 (Nrf2) regulated by Kelch-like ECH-associated protein 1 (Keap1) moderates the expression of antioxidant proteins and phase II detoxification enzymes to protect against oxidative stress-induced cell death and tissue injury (Kitakaze et al., 2019). Recent studies indicate that DJ-1 plays an indispensable role in Nrf2 stabilization and ubiquitination (Clements et al., 2006). Without intact DJ-1, the Nrf2 protein becomes unstable, and the activity of Nrf2-mediated downstream antioxidant enzymes is thereby suppressed (Zhang et al., 2017). Our previous studies have demonstrated that IPo application before reperfusion can reduce intestinal IR injury by activating Nrf2 pathway (Chen et al., 2020) and that N-acetylcysteine alleviates diabetic myocardial IR injury by promoting DJ-1 (Li et al., 2020). Here, we extend our previous studies by investigating the protective effects of IPo on myocardial IR-induced intestinal injury and the underlying mechanisms, with a focus on the role of DJ-1/Nrf2 signaling activation in rats.

## Materials and Methods

The animal study was reviewed and approved by the Laboratory Animal Welfare and Ethics Committee (IACUC) of Wuhan University in accordance with the Guidelines for the Care and Use of Laboratory Animals by the National Institutes of Health (NIH Publication No. 80-23), Directive 2010/63/EU in Europe and China.

### Animals

SPF-grade male Sprague-Dawley rats (Hunan Slac JD Laboratory Animal Co., Ltd., Hunan, China) weighing 200 ± 10 g were housed in individual cages in a climate-controlled room (23 ± 1°C; relative humidity 50 ± 10%) with 12h light–dark cycles and with free access to food and water. The rats were acclimated for 2 weeks before the experiments at the Animal Experiment Center of Renmin Hospital at Wuhan University.

### Experimental Protocol

**FIGURE 1:**
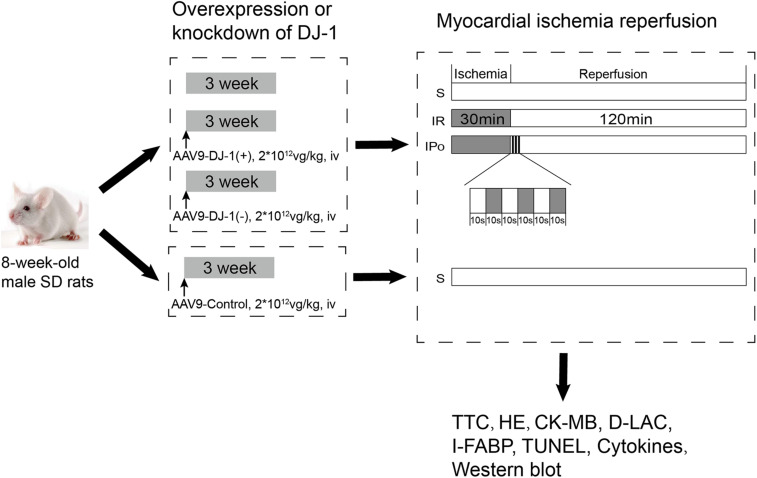
The schematic illustration of animal grouping and the experimental protocols. Animals were randomly treated with a mock treatment (control), AAV9-control, AAV9-DJ-1(+), or AAV9-DJ-1(–) before the establishment of the IR model. The AAV9-control group was subjected to only the sham operation as described in the figure, and then, the animals in the remaining groups, including the animals treated with a mock treatment, AAV9-DJ-1(+), or AAV9-DJ-1(–) were randomly allocated into three subgroups: (1) sham (S), (2) IR, and (3) IPo. A total of 60 rats in 10 groups were used in the experiments. The groupings and treatments (*n* = 6/group) are shown in the figure.

Animals were randomly treated with a mock treatment (control), AAV9-control, AAV9-DJ-1(+), or AAV9-DJ-1(−) before the establishment of the IR model. The AAV9-control group was subjected to only the sham operation as described in Figure 1, and then, the animals in the remaining groups, including the animals treated with a mock treatment, AAV9-DJ-1(+), or AAV9-DJ-1(−), were randomly allocated into three subgroups: (1) sham (S), (2) IR, and (3) IPo. A total of 60 rats in 10 groups were used in the experiments. The groupings and treatments (*n* = 6/group) are shown in Figure 1.

### Adeno-Associated Virus Infection

The model of DJ-1 overexpression and knockdown was established with adeno-associated virus serotype 9 (AAV9) vectors (Hanbio Biotechnology Co., Ltd., Shanghai, China). AAV9 vectors were produced by Hanbio Biotechnology Co. using the AAV-293 cell line via a modified 3-plasmid cotransfection method. AAV9-control, AAV9-DJ-1(+), or AAV9-DJ-1(−) was administered via tail vein injection at a dose of 2 × 10^12^ vg/kg 3 weeks before the myocardial IR induction was provided.

### Myocardial IR Model

All animals were fasted but had free access to water for 12 h before the experiments. We used the model of myocardial IR injury as described previously (Qiu et al., 2017, 2021; Zhou et al., 2017; Li et al., 2020) in which the rats were anesthetized completely and subjected to myocardial IR by clamping the left anterior descending coronary artery (LAD) for 30 min followed by reperfusion for 120 min. A ligature was placed ∼2 mm below the branch of the LAD with a 6-0 lesion-free line for ligation. IPo was implemented before reperfusion, which was performed by 3 cycles of 10 s of reocclusion and 10 s of reperfusion at the onset of reperfusion after 60 min of LAD occlusion (Zhou et al., 2017). Sham-operated rats underwent the same thoracotomy operation without LAD occlusion. The needle electrode was inserted under the limbs of the rat to monitor the ECG during the entire procedure. Ischemia was confirmed by the discoloration of the ischemic region. According to the 2013 AVMA Guidelines for the Euthanasia of Animals (Leary et al., 2020), rats were euthanized by intraperitoneal injection of pentobarbital sodium 250 mg/kg at the end of reperfusion. Tissues and blood were harvested and processed for biochemical analysis. The measurement was repeated three times, and the average of the values was then used for further analyses.

### Infarct Size Determination

Infarct size was measured by 3% Evans Blue dye (Sigma-Aldrich, Shanghai, China) and 1% 2,3,5-triphenyltetrazolium chloride (Sigma-Aldrich, Shanghai, China) staining as described previously (Xue et al., 2016). The stained myocardial slices were scanned (Epson v30) and assessed using an image analysis system (Image-Pro Plus 3.0, Media Cybernetics). The results were expressed as the percentage of infarcted myocardium area to the area of ischemic myocardium (IA/AAR%).

### Myocardial Creatine Kinase-MB Assay

Creatine kinase-MB (CK-MB) is a commonly used specific indicator of myocardial injury. Blood samples were collected at the end of reperfusion and centrifuged at 3,000 g for 15 min at 4°C. The serum was separated and stored at −20°C. The CK-MB levels were measured via enzyme immunoassay using a commercial ELISA kit (Nanjing Jiancheng Biocompany, Nanjing, China).

### Analysis of Intestinal Edema

Tissue edema was detected by the wet/dry weight (W/D) ratio of the gut. At the end of the experiments, 1 cm of small intestine without adipose tissue was collected from the same site in each animal, weighed, and placed in a drying oven at 80°C for 24 h. After drying, the specimens were reweighed, and the ratio of the weight before and after drying was calculated.

### Histopathology Assessment of Intestinal Tissue

Samples were collected from the same part of the small intestines at the distal end of ileum. All specimens were fixed with 4% paraformaldehyde solution and embedded in paraffin. Subsequently, the paraffin-embedded tissues were cut into 4 μm sections and assessed by hematoxylin and eosin (H&E) staining under a light microscope (original magnification 200×, Olympus BX50; Olympus Optical, Tokyo, Japan). Intestinal mucosal damage was evaluated using the improved Chiu’s score (Chiu et al., 1970): grade 0, normal mucosa; grade 1, development of subepithelial Gruenhagen’s space at the tip of the villus; grade 2, extension of the space with moderate epithelial lifting; grade 3, massive epithelial lifting with a few denuded villi; grade 4, denuded villi with dilated capillaries; and grade 5, disintegration of the lamina propria, ulceration, and hemorrhage. Higher scores were interpreted to indicate more severe damage.

### Evaluation of Intestinal Barrier Function

D-LAC and I-FABP in the serum were determined using ELISA kits (Nanjing Jiancheng Biocompany, Nanjing, China) in accordance with the instructions of the manufacturer.

### Cytokine Levels in the Serum and Intestinal Tissues

IL-1β, IL-10, and TNF-α levels in the serum and intestinal tissues were quantified using ELISA kits specific for mouse cytokines. All steps were performed according to the instructions of the manufacturer.

### Apoptosis Evaluation in Intestine by Terminal Deoxynucleotidyl Transferase-Mediated 2′-Deoxyuridine 5′-Triphosphate-Biotin Nick End Labeling (TUNEL) Assay

The 4 μm paraffin embedded sections were deparaffinized in xylene and double diluted water. The sections were then treated with proteinase K for 20 min at room temperature and subsequently incubated with a mixture of fluorescent labeling solution and TdT enzyme for 1 h in a humidified atmosphere. After washing with phosphate-buffered saline (PBS) and drying, the sections were incubated with DNase I for 10 min in a humidified atmosphere at room temperature. DAPI (Invitrogen; Thermo Fisher Scientific, Inc.) was used to stain the nuclei. The fluorescein isothiocyanate-labeled TUNEL-positive cells were imaged using fluorescence microscopy, and the apoptotic cells in the tissue section showed red fluorescence. The average number of apoptotic cells was calculated from five random fields with Image-Pro Plus software (version 6.0; Media Cybernetics, Rockville, MD, United States).

### Evaluation of Malondialdehyde (MDA) Content, Superoxide Dismutase (SOD) Activity, and GSH/GSSG

The MDA content, SOD activity, and GSH/GSSG of the serum and the intestine were tested by the appropriate assay kit (Nanjing Jiancheng Biocompany, Nanjing, China) according to the instructions of the manufacturer.

To determine the MDA content, the tissue was homogenized on ice in MDA lysis buffer containing BHT (100×), vortexed, and centrifugated. Then, 600 μL thiobarbituric acid (TBA) solution was added to each vial containing the standard and sample to form MDA-TBA adducts. The samples were incubated at 95°C for 60 min, cooled quickly to room temperature using an ice bath for 10 min, mixed with 1-butanol and 5 M NaCl with each reaction mixture, vortexed, and centrifuged at 16,000 × g for 3 min at room temperature. Then, 200 μL from each reaction mixture and sample was pipetted into a 96-well plate for analysis. Fluorescence intensity (λex = 532/λem = 553 nm) was measured.

To determine SOD activity, the tissue was mixed with a corresponding volume of PBS (generally at a weight/volume ratio of 1:9) in a glass homogenizer and ground thoroughly on ice. Finally, the homogenate was centrifuged at 5,000 rpm for 10 min, and the supernatant was collected as the sample to be tested. The samples were mixed with working solution thoroughly and incubated at 37°C for 20 min, and the absorbance was read at 450 nm using a microplate reader. SOD activity (inhibition rate%) was calculated using the following equation: {[(A positive control - A blank 1) - (A sample – A blank 2)]/(A positive control - A blank 1)} × 100.

To determine the GSH/GSSG ratio, the intestines of mice were homogenized in ice-cold 0.01 M HCl and centrifuged at 14,000 g for 10 min at 4°C. For total glutathione (GSH + GSSG), triethanolamine was added to the supernatant to give a final concentration of 6% (vol/vol). For GSSG measurements, 2% (vol/vol; final concentration) 2-vinylpyridine was also added. The assay buffers contained 1.52 mM NaH_2_PO_4_, 7.6 mM Na_2_HPO_4_, 0.485 mM EDTA, 1 U/mL glutathione reductase, and 0.1 mM NADPH (pH 7.5). After the addition of an aliquot of the sample, the assay mixture was incubated for 2 min. The reaction was started by adding 5,5′-dithiobis-(2-nitrobenzoic acid) to give a final concentration of 0.4 mM. The glutathione concentration was determined spectrophotometrically at a wavelength of 412 nm. The GSH content was calculated as the difference between the total glutathione and GSSG contents.

### Nuclear Protein Extraction

Nuclear and cytoplasmic proteins were extracted from the frozen intestinal tissues with a nuclear extraction kit (Beyotime Institute of Biotechnology, Haimen, China) according to the instructions of the manufacturer as described previously (Chen et al., 2020). Briefly, tissue (60 mg) from each rat was weighed, cut into small pieces, and homogenized in 200 μL cold lysis buffer. After homogenization, the cytoplasmic fraction was collected by two centrifugations at 4°C, 1,500 × g and 12,000 × g for 5 min, and the pellet was resuspended in nuclear isolation buffer. Then, the nuclear fraction was obtained from the suspension by centrifugation at 16,000 × g for 10 min. The cytoplasmic and nuclear fractions were transferred to clean microcentrifuge tubes, aliquoted, and stored at −80°C until the time of the assay. To estimate the amount of each fraction within the liver, proteins were determined using the BCA (bicinchoninic acid) Protein Assay kit (Thermo Scientific).

### Western Blot Analysis

Tissues were weighed, lysed in RIPA buffer (1% NP40, 0.5% sodium deoxycholate, and 0.1% SDS in PBS), and homogenized at 4°C using a TissueRuptor (QIAGEN, Hilden, Germany). The total protein concentration of the supernatant was determined by a BCA Protein Assay Reagent. Samples were loaded on 12% [for Occludin, Bax, Bcl-2, Caspase-3, Keap-1, Heme Oxygenase-1 (HO-1), NAD(P)H:quinone oxidoreductase 1 (NQO1), and β-actin] or 8% (for Nrf2 and Lamin B1) sodium dodecyl sulfate polyacrylamide gel electrophoresis (SDS-PAGE) at 100 V. After electrophoresis, the proteins were transferred onto polyvinylidene fluoride (PVDF) membranes (Thermo Fisher Scientific, Inc., Waltham, MA, United States) at 200 mA. The membranes were blocked for 2 h with 5% BSA (Bio-Rad Laboratories, Hercules, CA, United States) in TBS (10 mM Tris and 100 mM NaCl) and incubated overnight at 4°C with rabbit anti-mouse antibodies against DJ-1, Occludin, Bax, Bcl-2, Caspase-3, Nrf2, HO-1, NQO-1, β-actin (1:1,000), and Lamin B1 (1:200). After washing three times with TTBS, the membranes were incubated with the corresponding LI-COR IRDye800CW goat anti-rabbit secondary antibody (1:10,000) (Li-Cor Bioscience, Lincoln, NE, United States) for 1 h at room temperature. The intensity of the identified bands was detected using the Odyssey two-color infrared laser imaging system, and densitometry was carried out using Odyssey software (both from Li-Cor Bioscience, Lincoln, NE, United States).

### Statistics Analysis

Data were expressed as dot plot and mean ± SD or median and range with Box-Whisker’s plot wherever appropriate and were analyzed with one-way analysis of variance (ANOVA), followed by Tukey’s or Dennis’s *post hoc* test (GraphPad Prism 8.0, San Diego, CA, United States). The effects of AAV-DJ-1(+) or AAV-DJ-1(−) in the presence of IPo were analyzed by two-way ANOVA with repeated measures (one between factor and one within factor), followed by Tukey’s multiple comparison testing with GraphPad Prism 8.0. A *P*-value of 0.05 or less was considered to be a significant difference.

## Results

### Ischemic Postconditioning Attenuated the Myocardial IR-Induced Myocardial Damage and Intestinal Barrier Injury

**FIGURE 2:**
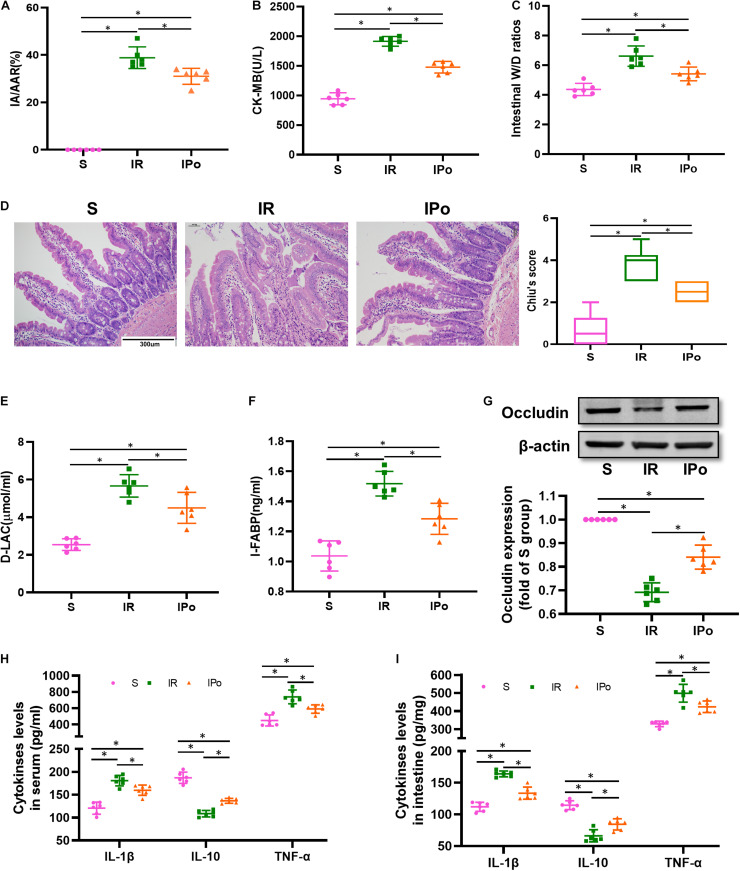
Ischemic postconditioning alleviated myocardial IR-induced myocardial and intestinal injury. **(A)** Infarct area relative to the area at risk (IA/AAR × 100%). **(B)** Serum CK-MB level. **(C)** Intestinal water W/D ratios. **(D)** Histopathologic changes of the small-intestinal mucosa under light microscopy imaging (H&E staining, Scale bar = 300 μm). Intestinal mucosa injury was graded by Chiu’s score. Serum concentrations of D-LA **(E)** and I-FABP **(F)**. **(G)** Expression of occludin. The levels of inflammatory cytokines (IL-1β, IL-10, and TNF-α) in the serum **(H)** and intestine **(I)**. β-actin were detected as loading control. S, Sham; IR, ischemia reperfusion; IPo, ischemic postconditioning. Data are mean ± SD or Box-Whisker’s plot (*n* = 6); *P < 0.05.

To confirm the protective effects of IPo, we evaluated the myocardial and intestinal injury induced by myocardial IR with or without IPo. As shown in Figure 2A, myocardial IR induced post-ischemia myocardial infarction, and IPo significantly decreased infarct sizes compared with the IR group (*P* < 0.05). We subsequently measured the levels of biochemical markers of myocardial injury, and the results showed that CK-MB levels were significantly increased in IR group compared with the S group and the IPo noticeably reduced CK-MB (Figure 2B).

The intestinal W/D ratio, an indicator of damage to intestinal permeability, was significantly elevated in the IR group compared with the S group and decreased significantly after IPo treatment (Figure 2C, *P* < 0.05). Myocardial IR induced edematous, severed, or denuded intestinal villi, and the gap between epithelial cells was significantly increased (*P* < 0.05). However, a significant amelioration of histological injury was observed after received IPo treatment (Figure 2D, *P* < 0.05). Furthermore, the serum concentrations of D-LA (Figure 2E) and I-FABP (Figure 2F) were used as biomarkers to estimate the function of the intestinal epithelium. Accordingly, D-LA and I-FABP levels were significantly increased in the IR group relative to those in the S group. The IPo treatment significantly decreased the IR-induced D-LA and I-FABP increases. Tight junctions are the fundamental features of both the epithelium and the endothelium and are indispensable for organ formation and homeostasis. Myocardial IR disrupted the expression of intestinal epithelial tight junction protein occludin, and IPo restored the expression of occludin to normal levels (Figure 2G). Expression levels of the proinflammatory factor IL-1β, TNF-α, and the anti-inflammatory factor IL-10 were measured in the serum and intestine. TNF-α and IL-6 levels increased, and IL-10 level decreased markedly in the IR group, and these changes were significantly attenuated by IPo treatment (Figures 2H,I, *P* < 0.05).

### Ischemic Postconditioning Ameliorated the Myocardial IR-Induced Intestinal Apoptosis and Suppressed the Oxidative Stress

**FIGURE 3:**
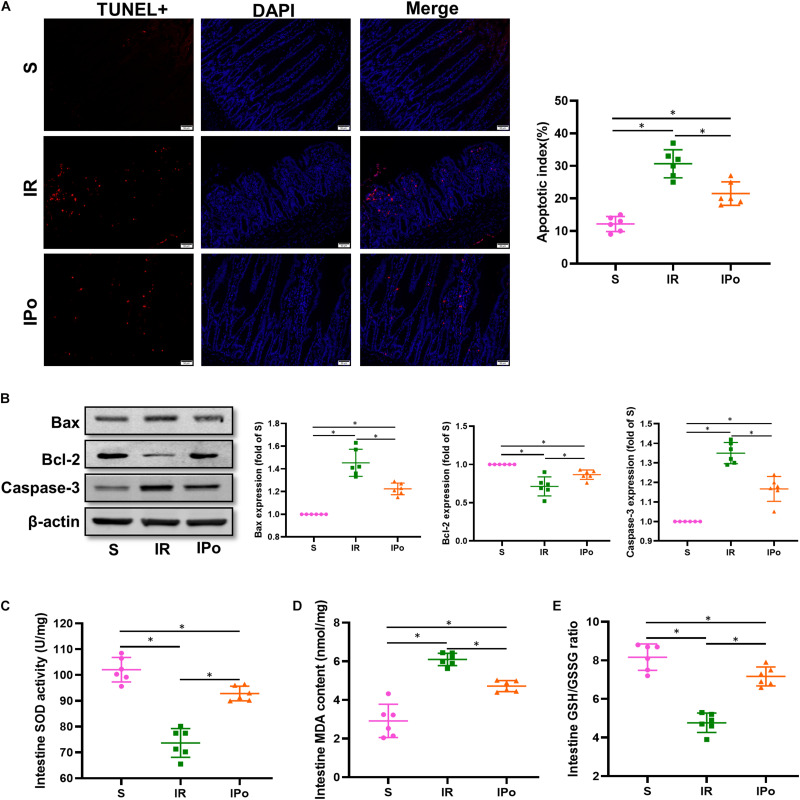
Ischemic postconditioning ameliorates myocardial IR-induced apoptosis and suppresses oxidative stress. **(A)** The TUNEL staining for cell apoptosis rates. **(B)** Expression of apoptosis-related proteins Bax, Bcl-2, and caspase-3. **(C)** SOD. **(D)** MDA. **(E)** GSH/GSSG ratio. β-actin was detected as loading control, and β-action gel is the same as in Figure 2. S, Sham; IR, ischemia reperfusion; IPo, ischemic postconditioning. Data are mean ± SD (*n* = 6); *P < 0.05.

As shown in Figure 3A for TUNEL staining of the intestine, the cell apoptosis rate in the IR group increased compared with that in the S group (*P* < 0.05). Compared with the IR group, the cell apoptosis rate in the IPo group decreased (*P* < 0.05). Bcl-2 protein levels in the IR group decreased (*P* < 0.05), and Bax and caspase-3 levels increased compared with those in the S group (*P* < 0.05). IPo memorably increased the Bcl-2 protein level and decreased the expression of Bax and caspase-3 (Figure 3B, *P* < 0.05). Myocardial IR treatment dramatically decreased the activity of SOD and the GSH/GSSG ratio. Conversely, IPo significantly enhanced the SOD activity (Figure 3C) and the GSH/GSSG ratio (Figure 3E) compared to those in the IR group (*P* < 0.05). The levels of MDA increased significantly in the IR group versus the S group. Interestingly, IPo significantly decreased the IR-induced increase in MDA content (Figure 3D, *P* < 0.05).

### DJ-1/Nrf2 is Crucial in the Protection of IPo Against Myocardial IR-Induced Intestinal Injury

**FIGURE 4:**
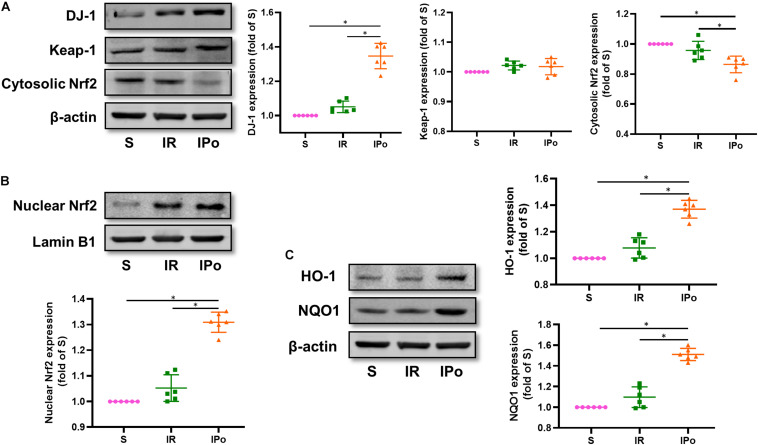
Ischemic postconditioning activates DJ-1, induces Nrf2 nuclear translocation, and upregulates HO-1 and NQO1 expression. **(A)** Expression of DJ-1, Keap-1, Cytosolic Nrf2. **(B)** Expression of Nrf2 in the nucleus and **(C)** of its downstream targets HO-1 and NQO1. Lamin B1 and β-actin were detected as loading controls, respectively, and β-action gel is the same as in Figure 2. S, Sham; IR, ischemia reperfusion; IPo, ischemic postconditioning. Data are presented as the mean ± SD (*n* = 6). *P < 0.05.

Compared with that in the S group, the expression of DJ-1 and the accumulation of Nrf2 protein in the nucleus were slightly increased in the IR group and were clearly enhanced in the IPO group (Figure 4A, *P* < 0.05). Consequently, the protein expression of two downstream targets of Nrf2, HO-1 and NQO1, was remarkably increased in the IPO group compared with the IR group (Figure 4B, *P* < 0.05).

### Effects of DJ-1 Overexpression on IPo-Induced Intestinal Protection Against Myocardial IR

To determine whether DJ-1 overexpression or knockdown affects IPo-induced intestinal protection, DJ-1 was overexpressed or knocked down by intravenous injection of AAV. The results showed that at 3 weeks after AAV-Control and AAV-DJ-1 infection, there was no difference in IA/AAR, CK-MB, Chiu’s score, intestinal wet/dry ratio, D-LAC, and I-FABP in the S group treated with or without AAV-Control, AAV-DJ-1(+), and AAV-DJ-1(−) (Supplementary Figures 1A–F). In addition, there was no significant difference in the DJ-1 protein expression in the S group treated with or without AAV-control (Supplementary Figure 1G).

**FIGURE 5:**
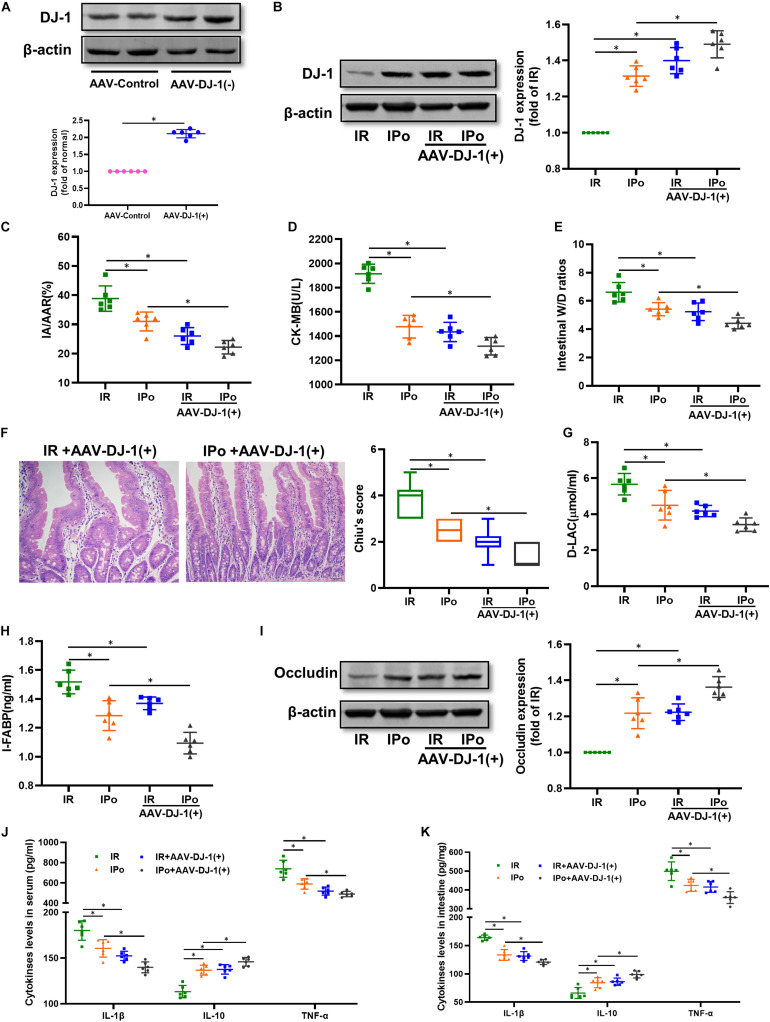
Overexpression of DJ-1 promoted IPo-induced protection against myocardial IR-induced intestinal injury. **(A,B)** Expression of DJ-1 level. **(C)** Infarct area relative to the area at risk (IA/AAR × 100%). **(D)** serum CK-MB level. **(E)** Intestinal water W/D ratios. **(F)** Histopathologic changes of the small-intestinal mucosa under light microscopy imaging (H&E staining, Scale bar = 300 μm). Intestinal mucosa injury was graded by Chiu’s score. Serum concentrations of D-LA **(G)** and I-FABP **(H)**. **(I)** Expression of occludin. The levels of inflammatory cytokines (IL-1β, IL-10, and TNF-α) in the serum **(J)** and intestine **(K)**. β-actin was detected as loading control. S, Sham; IR, ischemia reperfusion; IPo, ischemic postconditioning. Data are mean ± SD or Box-Whisker’s plot (*n* = 6); *P < 0.05.

At 3 weeks after AAV-DJ-1(+) infection, we determined that the expression of DJ-1 in AAV-DJ-1(+) group rats was nearly twofold higher than that in the AAV-control group (Figure 5A and Supplementary Figure 1G). Compared to the IPo group, IPo + AAV-DJ-1(+) further increased the expression of DJ-1 (Figure 5B). As shown in Figures 5C–I, DJ-1 overexpression reduced the infarct sizes, CK-MB, intestinal W/D ratio, histological injury, D-LA, I-FABP, and upregulation of occludin expression similar to IPo treatment, whereas the combination of DJ-1 overexpression and IPo further markedly decreased the infarct sizes, CK-MB, intestinal W/D ratio, histological injury, D-LA, I-FABP, and upregulation of occludin, indicating that the DJ-1 overexpression facilitated IPo-induced intestinal protection. In the serum and intestine, TNF-α and IL-6 levels decreased and IL-10 levels increased in the IR + AAV-DJ-1(+) group compared with the IR alone, and these changes were significantly attenuated by combining IPo with AAV-DJ-1(+) (Figures 5J,K, *P* < 0.05).

### Effects of the DJ-1 Overexpression on IPo-Mediated Amelioration of Myocardial IR-Induced Intestinal Apoptosis, Suppression of Oxidative Stress, and Activation of Nrf2

**FIGURE 6:**
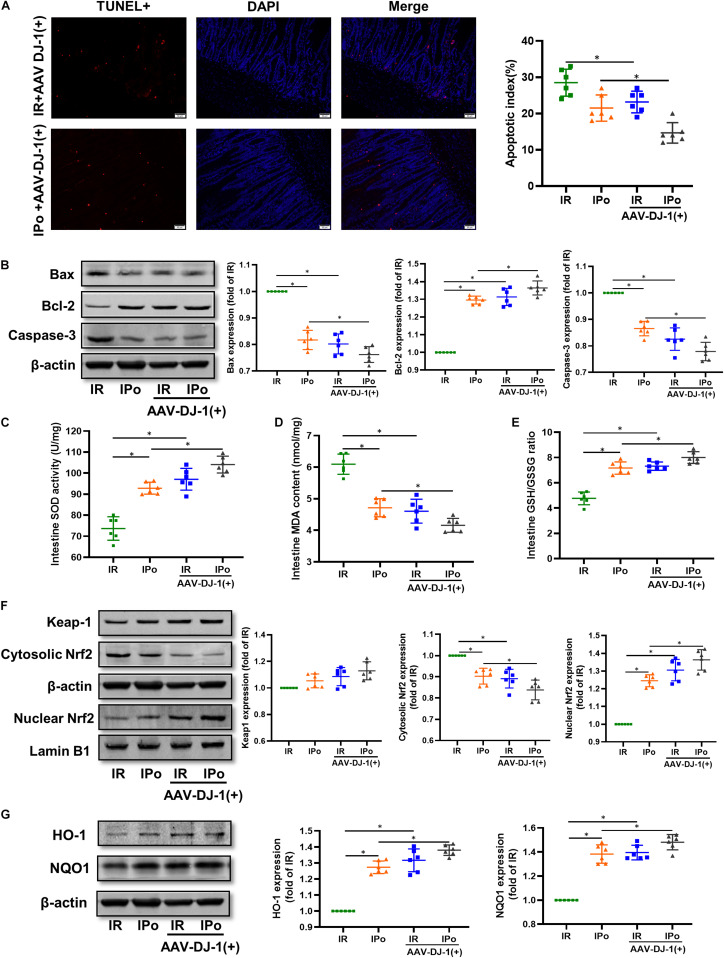
Overexpression of DJ-1 promoted IPo-induced protection via ameliorated myocardial IR-induced apoptosis and suppressed oxidative stress by regulating Nrf2 pathway. **(A)** The TUNEL staining for cell apoptosis rates. **(B)** Expression of apoptosis-related proteins Bax, Bcl-2, and caspase-3. **(C)** SOD. **(D)** MDA. **(E)** GSH/GSSG ratio. **(F)** Expression of Keap-1 and Nrf2 in the cytosolic and nucleus, **(G)** Expression of HO-1 and NQO1. Lamin B1 and β-actin were detected as loading controls, respectively, and β-actin gel is the same as in Figure 5. S, Sham; IR, ischemia reperfusion; IPo, ischemic postconditioning. Data are presented as the mean ± SD (*n* = 6). *P < 0.05.

As shown in Figure 6A for TUNEL staining of the intestine, the overexpression of DJ-1 decreased cell apoptosis in a way similar to IPo treatment, whereas the combination of DJ-1 overexpression and IPo further markedly decreased cell apoptosis. Bcl-2 protein in the IR + AAV-DJ-1(+) group increased (*P* < 0.05), and Bax and caspase-3 levels decreased compared with those in the IR group (*P* < 0.05). IPo + AAV-DJ-1(+) memorably increased the Bcl-2 protein level and decreased the expression of Bax and caspase-3 (Figure 6B, *P* < 0.05). IR + AAV-DJ-1(+) significantly enhanced the SOD activity (Figure 6C) and GSH/GSSG (Figure 6E) when compared to the IR group, and IPo + AAV-DJ-1(+) further enhanced the SOD (Figure 6C) and the GSH/GSSG ratio (Figure 6E, *P* < 0.05). The levels of MDA increased significantly after IR + AAV-DJ-1(+) versus IR. Interestingly, the combination of DJ-1 overexpression and IPo further decreased the IR-induced increase in MDA content (Figure 6D, *P* < 0.05). Compared with that in the IR group, the accumulation of Nrf2 in the nucleus was dramatically increased in the IR + AAV-DJ-1(+) group (Figure 6F, *P* < 0.05). Consequently, the protein expression of two downstream targets of Nrf2, HO-1, and NQO1 was remarkably increased in the IR + AAV-DJ-1(+) group compared with the IR group (Figure 6G, *P* < 0.05). IPo + AAV-DJ-1(+) prominently upregulated the expression of nuclear Nrf2, HO-1, and NQO1.

### Knockdown of DJ-1 Abrogated IPo-Induced Intestinal Protection Against Myocardial IR

**FIGURE 7:**
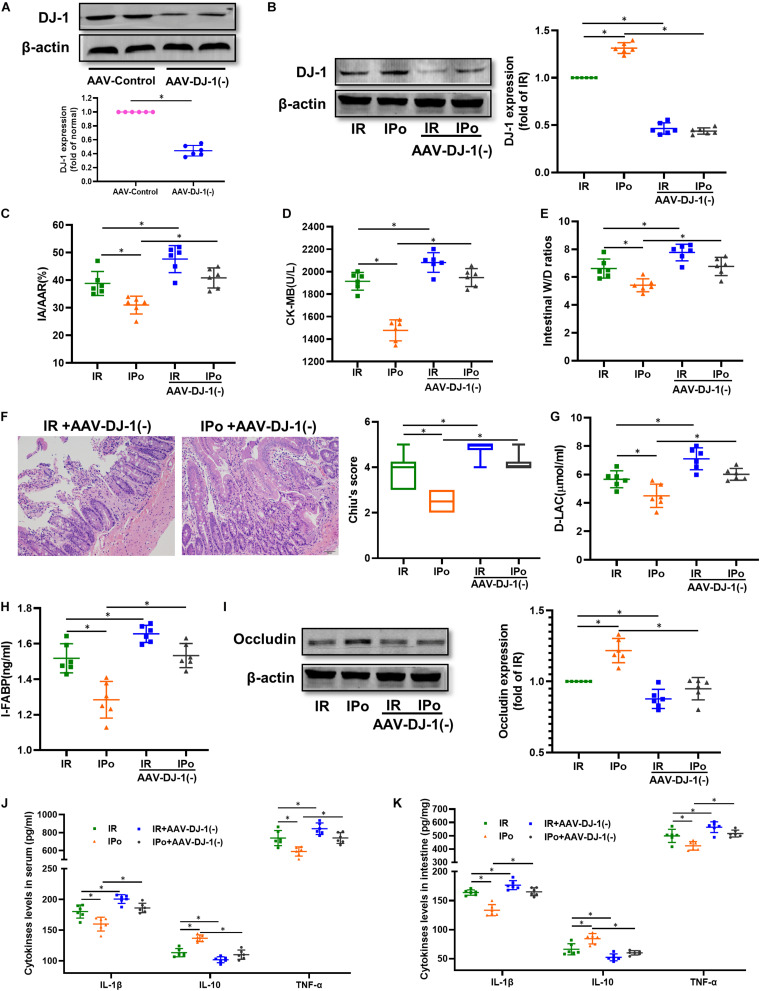
Knockdown of DJ-1 abolished IPo induced protection against myocardial IR-induced intestinal injury. **(A,B)** Expression of DJ-1 level. **(C)** Infarct area relative to the area at risk (IA/AAR × 100%). **(D)** serum CK-MB level. **(E)** Intestinal water W/D ratios. **(F)** Histopathologic changes of the small-intestinal mucosa under light microscopy imaging (H&E staining, Scale bar = 300 μm). Intestinal mucosa injury was graded by Chiu’s score. Serum concentrations of D-LA **(G)** and I-FABP **(H)**. **(I)** Expression of occludin. The levels of inflammatory cytokines (IL-1β, IL-10, and TNF-α) in the serum **(J)** and intestine **(K)**. β-actin was detected as loading control. S, Sham; IR, ischemia reperfusion; IPo, ischemic postconditioning. Data are mean ± SD or Box-Whisker’s plot (*n* = 6); *P < 0.05.

We knocked down the DJ-1 protein via AAV9-DJ-1 injection. At 3 weeks after AAV-DJ-1(−) infection, the expression of the DJ-1 protein in the AAV-DJ-1(−) group was less than half of that in the AAV-control group (Figure 7A). Knockdown of DJ-1 abolished the increased protein expression of DJ-1 induced by IPo (Figure 7B). As shown in Figures 7C–I, knockdown of DJ-1 aggravated IR injury; abolished IPo-induced protection; displayed increased infarct sizes, CK-MB levels, intestinal W/D ratios, Chiu’s score, D-LA, and I-FABP; and downregulated occludin expression in the IR + AAV-DJ-1(−) and IPo + AAV-DJ-1(−) group compared with IR and IPo alone, respectively. TNF-α and IL-6 levels increased and IL-10 levels decreased in the IPo + AAV-DJ-1(−) group compared with IPo alone (Figures 7J,K, *P* < 0.05).

### Knockdown of DJ-1 Abolished the IPo-Induced Protection, Increased the Intestinal Apoptosis, and Activated the Oxidative Stress

**FIGURE 8:**
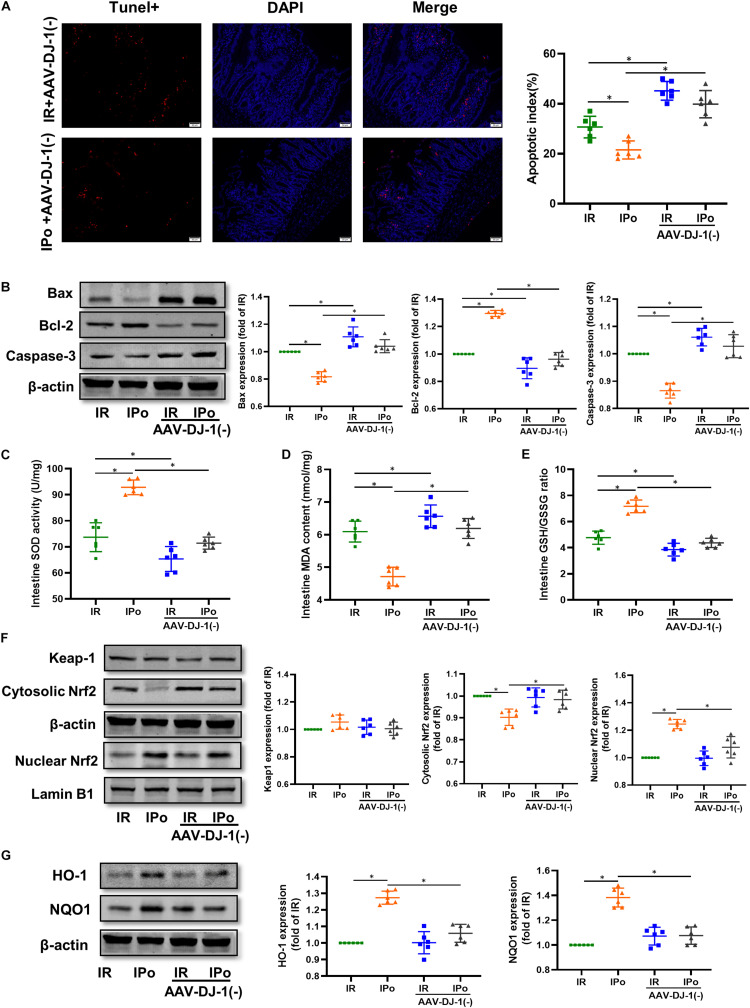
Knockdown of DJ-1 abrogated IPo-induced protection via ameliorated myocardial IR-induced apoptosis and suppressed oxidative stress by regulating Nrf2 pathway. **(A)** The TUNEL staining for cell apoptosis rates. **(B)** Expression of apoptosis-related proteins Bax, Bcl-2 and Caspase-3. **(C)** SOD. **(D)** MDA. **(E)** GSH/GSSG ratio. **(F)** Expression of Keap-1, Nrf2 in the cytosolic and nucleus, **(G)** Expression of HO-1 and NQO1. Lamin B1, β-action were detected as loading controls, respectively, and β-action gel is the same as in Figure 7. S, Sham; IR, ischemia reperfusion; IPo, ischemic postconditioning. Data are presented as the mean ± SD (*n* = 6). *P < 0.05.

As shown in Figure 8A for TUNEL staining of the intestine, knockdown of DJ-1 by AAV aggravated the cell apoptosis induced by IR alone and abolished IPo-induced intestinal epithelial cell protection. Bcl-2 protein levels in the IR + AAV-DJ-1(−) group decreased (*P* < 0.05), and Bax and caspase-3 levels increased compared with those in the IR group (*P* < 0.05). IPo + AAV-DJ-1(−) decreased the Bcl-2 protein level, increased the expression of Bax and caspase-3, and abolished the IPo-induced protection (Figure 8B, *P* < 0.05). IR + AAV-DJ-1(−) further reduced the SOD activity (Figure 8C) and the GSH/GSSG ratio (Figure 8E), and increased the MDA content (Figure 8D) compared with those observed after IR, while IPo + AAV-DJ-1(−) reversed the increased SOD activity and the GSH/GSSG ratio, and decreased the MDA content caused by IPo (Figures 8C–E, *P* < 0.05). Compared to that in the IPo group, the accumulation of Nrf2 in the nucleus was dramatically decreased in the IPo + AAV-DJ-1(−) group (Figure 8F, *P* < 0.05). Consequently, the protein expression of two downstream targets of Nrf2, HO-1, and NQO1, remarkably decreased in the IPo + AAV-DJ-1(−) group compared with the IPo group (Figure 8G, *P* < 0.05). IPo + AAV-DJ-1(−) offsets the increased expression of nuclear Nrf2, HO-1, and NQO1.

## Discussion

Here we demonstrate the presence of overt mucosal barrier dysfunction in the rat small intestine following myocardial IR, with marked histopathological alterations, increased serum biomarkers, and decreased expression of tight junction protein occludin. Our study also found that DJ-1/Nrf2 was activated in the rat small intestine by IPo, and subsequently, it could attenuate myocardial IR-induced intestinal injury. In addition, knockdown of DJ-1 abolished the gut-protective effects of IPo, while DJ-1 overexpression had protective effects similar to those of IPo on myocardial IR-induced intestinal injury. These results suggest that the DJ-1/Nrf2 activation by IPo likely contributes to protecting the intestine from myocardial IR.

The gut-heart axis as a novel concept in the field of heart research has recently been acknowledged in AMI. Cardiac dysfunction following AMI induces acutely adaptive but chronically maladaptive hemodynamic, neurohumoral, and proinflammatory responses, which all affect the intestine. A previous study demonstrated that the gut microbiota significantly changed and that the gut barrier was notably impaired at day 7 post AMI (Wu et al., 2017). Metabolites derived from the intestinal microbiota are linked to the severity of MI (Lam et al., 2016). The main possible mechanism of intestinal barrier dysfunction is hemodynamic changes in the intestines post AMI (Mao et al., 2011). The intestine is a blood-demanding organ, and villi (and microvilli) are prone to functional ischemia due to reduced blood flow (Takala, 1996). In the setting of low cardiac output following AMI, substantial hypoperfusion and congestion in the intestines can alter gut morphology, leading to an increase in gut permeability, intestinal dysfunction, and possibly translocation of gut microbiota (Nagatomo and Tang, 2015). All of these factors contribute to the pathogenesis of future cardiovascular events by inducing inflammation (Zhou et al., 2018). It is of paramount importance to rescue the ischemic myocardium and prevent intestinal damage after AMI. Prompt and successful restoration of blood flow with either thrombolytic agents or primary PCI is crucial to salvage ischemic myocardium, reduce infarct size, improve left ventricular function, and improve clinical outcomes (Kushner et al., 2009). However, abrupt reperfusion itself may lead to accelerated and additional myocardial injury beyond that generated by ischemia alone (Bulluck et al., 2016), and some damages are even irreversible. The pattern of injury that is inflicted on the myocardium has been termed IR injury (Hausenloy and Yellon, 2013), which has been described and characterized in numerous organs. Intestinal hypoperfusion and congestion caused by AMI combined with oxidative stress and inflammation induced by myocardial IR injury result in serious intestinal mucosal barrier injury, which, in turn, exacerbates AMI, leading to severe cardiovascular events and damage of other remote organs. Overt histopathological alterations in the structure and inflammation of the intestine can be observed as early as 2 h after myocardial IR, and changes were associated with increased activity of MMP-2 (László et al., 2020). The bacterial metabolites translocated into the blood circulation further amplify the systemic inflammatory response, increase the level of inflammatory mediators such as TNF-α, IL-1, and IL-6, and, in turn, aggravate intestinal mucosal injury (Seropian et al., 2016). Consistent with previous results, our results showed altered intestinal morphology such as denuded villi and disintegration of the lamina propria, increased serum biomarkers (including CK-MB, D-Lac, I-FABP), and decreased expression of tight junction protein occludin 2 h after reperfusion of the ischemic myocardium. In addition, levels of the proinflammatory factors IL-1β and TNF-α increased and levels of the anti-inflammatory factor IL-10 decreased in both the intestine and serum. Therapeutic intervention that protects gut function may be a potential option to improve cardiovascular outcomes after myocardial IR.

Ischemic conditioning, brief repeated episodes of IR, induces cardioprotection and reduces myocardial IR injury not only in experimental animal models but also in patients (Heusch, 2017; Stoian et al., 2019). Ischemic pre- and postconditioning both have the function of organic protection (Heusch, 2015). However, due to the fact that the time of myocardial ischemia of patients cannot be predicted, the application of ischemic preconditioning is relatively limited, and ischemic postconditioning (IPo) is more dominant and has more research prospects. IPo was established by the group of Vinten-Johansen (Zhao et al., 2003) who first reported 3 cycles of 30 s reperfusion/30 s reocclusion immediately at the onset of reperfusion after 60 min of LAD occlusion. A recent study showed that 3 cycles of 10 s of reocclusion and 10 s of reperfusion at the onset of reperfusion conferred cardioprotection by reducing ROS production through AMPK-independent activation of STAT3 at Ser727 (Zhu et al., 2020). The myocardial protective effect of IPo also relies on regulating energy homeostasis and the expression of myocardial mitochondrial proteins (Cao et al., 2016). Xu et al. (2011, p. 1) showed that IPo attenuated pulmonary neutrophil accumulation and activation and lung IR injury and reduced systemic inflammatory responses by activating HO-1. Our previous studies also confirmed that IPo reduced the serum expression of cytokines induced by intestinal IR and reduced intestinal IR-induced renal injury (Chen et al., 2020). In our study, we demonstrated that IPo performed by 3 cycles of 10 s of reocclusion and 10 s of reperfusion could effectively alleviate the pathological changes of intestinal tissue caused by myocardial IR, reduce the apoptosis of intestinal cells, decrease the intestinal inflammatory response, and partially restore intestinal function. A better understanding of the signal transduction underlying conditioning may help to recruit protection without the inevitable injury associated with IR.

DJ-1 is a promising biomarker and therapeutic target for PD, as well as a broader range of neurodegenerative diseases (Repici and Giorgini, 2019). As a multifunctional protein, DJ-1 might serve as a regulator of antioxidant and antiapoptosis activities. *In vitro* and *in vivo* models show that overexpression of DJ-1 decreased the expression of proapoptotic protein Bax and inhibited the expression of proapoptotic caspase-3 and caspase-9 proteins due to ROS production in mammalian cells by inhibiting p53 transcriptional activity (Fan et al., 2008). DJ-1 might improve the mitochondrial function and inhibit the ROS production to reduce apoptosis of retinal pericytes through phosphatidylinositol 3-kinase (PI3K)/Akt/mTOR to protect against high glucose induced-oxidative injury (Zeng et al., 2019). Overexpression of DJ-1 protects cells against oxidative stress-induced injury, whereas knockdown or knockout of DJ-1 increases the susceptibility to oxidative injury in models of cerebral ischemia and neuronal cell death (Yanagisawa et al., 2008). In the heart, DJ-1 has been reported to act as a mediator of hypoxic preconditioning in H9c2 rat myoblasts (Lu et al., 2012). Mice deficient in DJ-1 develop more severe heart failure in response to aortic banding (Billia et al., 2013). In response to IR injury, adult hearts lacking DJ-1 exaggerated myocardial injury, displayed increased areas of infarction, worsened left ventricular function (Shimizu et al., 2016), and were resistant to the endogenous cardioprotective phenomenon of IPC (Dongworth et al., 2014). Overexpression of DJ-1 can reduce ROS production and alleviate myocardial cell apoptosis by inhibiting PTEN/PI3K/Akt signaling pathway (Xin et al., 2019). Moreover, DJ-1 deficiency significantly aggravated inflammatory bowel disease, evidenced by increased intestinal inflammation and exacerbated intestinal epithelial cell apoptosis (Zhang et al., 2020). si-DJ-1 attenuated the cytoprotective effect of protocatechuic acid on ketoprofen-induced mitochondrial oxidative stress and apoptotic cell death (Cheng et al., 2019). In this study, we found that the expression of intestinal DJ-1 slightly increased after myocardial IR, while IPo further activated DJ-1, thereby reducing MDA, reducing the expression of Bax and caspase-3, increasing antioxidant capacity, and, finally, alleviating intestinal barrier injury caused by myocardial IR.

Nrf2 is a key regulator of cellular reduction–oxidation homeostasis. Upon exposure to various pathophysiologic stresses, Nrf2 dissociates from Keap1 upon phosphorylation at specific Nrf2 serine and/or threonine residues through the activation of several upstream kinases. Then, it translocates to the nucleus, where it binds antioxidant response element (ARE) sequences, thus resulting in the transcriptional activation of antioxidant genes, such as HO-1 and NQO-1 (Wang et al., 2016). In the previous study, we confirmed that the Nrf2/HO-1 signaling pathway activated by IPo likely alleviated the intestinal IR-induced renal injury (Chen et al., 2020). Our data showed that IPo strongly induced the Nrf2 translocation and increased the downstream antioxidant enzymes in the intestine. Moreover, IPo markedly alleviated the oxidative stress and reversed the impaired activity of SOD. Recent reports also suggest that DJ-1 is critical for the modulation of the bioactivity of Nrf2, which affects the response to antioxidant enzymes (Zhang et al., 2017). It has been confirmed that DJ-1 is essential for Nrf2 stabilization by preventing the association of Nrf2 with Keap1, an inhibitor protein that boosts Nrf2 ubiquitination and degradation (Vavougios et al., 2018). DJ-1 overexpression enhanced the expression of Nrf2 and its downstream phase II metabolic enzymes, including HO-1, NQO1, GCLC, and GCLM. In contrast, when Nrf2 was inhibited, even if DJ-1 was overexpressed, the expression of phase II metabolic enzymes was not significantly increased, and rat retinal pericyte apoptosis was not significantly reduced (Wang et al., 2020). Protocatechuic acid protected gastrointestinal mucosa cells against ketoprofen-induced oxidative stress by overexpressing DJ-1/Nrf2, while increased antioxidant enzyme expression could be blocked by si-DJ-1 (Cheng et al., 2019). Similarly, our results indicated that IPo effectively prevented myocardial IR-induced intestinal injury through induction of the DJ-1/Nrf2 pathways. The increased expression of DJ-1 induced by IPO can promote Nrf2 to translocation into the nucleus, increase the expression of downstream HO-1 and NQO1 proteins, reduce cell apoptosis, and alleviate intestinal barrier damage caused by myocardial IR. The AAV-9-mediated overexpression of DJ-1 can achieve the same effect as IPo. Knockdown of DJ-1 eliminated the protective effect of IPo on the intestine against myocardial IR, as evidenced by increased intestinal oxidative stress and exacerbated intestinal epithelial cell apoptosis.

The implications of our current study may be enormous as organ crosstalk occurs all the time under pathophysiological conditions. This crosstalk can ultimately result in multiorgan injury or failure; therefore, preventive and/or treatment strategies are urgently needed to “tackle” remote organ injuries and improve long-term outcomes. Although our results show that IPo is of great value for preventing AMI and myocardial-induced intestinal injury, it is still challenging to translate this approach into clinical applications. This study also has some limitations. Our animal experiment used reductionist approaches and was performed in young and healthy rats that lacked the risk factors, comorbidities, and comedications which are the characteristics of patients suffering from AMI or undergoing PCI or cardiovascular surgery. Advanced age, diabetes, hypertension, and hypercholesterolemia all interfere with cardioprotective interventions and attenuate or abrogate infarct size reduction induced by IPo. In the future, our group will add relevant risk factors like diabetes to verify the protective effect of IPo.

In summary, our data demonstrated that IPo protects against the myocardial IR-induced intestinal injury. This beneficial effect was found to be closely associated with its ability to activate the DJ-1/Nrf2 signaling pathway which subsequently activates the antioxidants and reduces the intestinal mucosal cell apoptosis.

## Data Availability Statement

The original contributions presented in the study are included in the article/Supplementary Material, further inquiries can be directed to the corresponding author/s.

## Ethics Statement

The animal study was reviewed and approved by the Laboratory Animal Welfare and Ethics Committee (IACUC) of Wuhan University.

## Author Contributions

Q-tM and Z-yX contributed to conception and design of the study. RC, WL, and W-yL organized the database. QZ performed the statistical analysis. RC wrote the first draft of the manuscript. RC, YZ, ZQ, and KD wrote the sections of the manuscript. All authors contributed to manuscript revision, and read and approved the submitted version.

## Conflict of Interest

The authors declare that the research was conducted in the absence of any commercial or financial relationships that could be construed as a potential conflict of interest.

## Supplementary Material

Supplementary Figure 1 Verify the safety of AAV. **(A)** Infarct area relative to the area at risk (IA/AAR × 100%). **(B)** serum CK-MB level. **(C)** Intestinal water W/D ratios. Serum concentrations of D-LA **(D)** and I-FABP **(E)**. **(F)** Histopathologic changes of the small-intestinal mucosa under light microscopy imaging (H&E staining, Scale bar = 300 μm). Intestinal mucosa injury was graded by Chiu’s score. **(G)** Expression of DJ-1 level. S, Sham; IR, ischemia reperfusion; IPo, ischemic postconditioning. Data are mean ± SD or Box-Whisker’s plot (*n* = 6); ^∗^*P* < 0.05.

## Abbreviations

IR: ischemia reperfusion
AMI: acute myocardial infarction
IPo: ischemic postconditioning
MI: myocardial infarction
CVD: cardiovascular disease
HF: heart failure
PCI: percutaneous coronary intervention
ROS: reactive oxygen species
Nrf2: nuclear factor-erythroid 2-related factor 2
Keap1: Kelch-like ECH-associated protein 1
AAV9: recombinant adeno-associated virus serotype 9
LAD: left anterior descending coronary artery
CK-MB: creatine kinase-MB
W/D ratio: wet/dry weight ratio
H&E: hematoxylin and eosin
TUNEL: terminal deoxynucleotidyl transferase-mediated 2 ′ -deoxyuridine 5 ′ -triphosphate-biotin nick end labeling
MDA: malondialdehyde
TBA: thiobarbituric acid
SOD: superoxide dismutase
HO-1: heme oxygenase-1
NQO1: NAD(P)H quinone oxidoreductase 1
SDS-PAGE: sodium dodecyl sulfate polyacrylamide gel electrophoresis
PVDF: polyvinylidene fluoride
PI3K: phosphoinositide 3-kinase.
