# Supplementary figures and images for: Ischemic Postconditioning-Mediated DJ-1 Activation Mitigate Intestinal Mucosa Injury Induced by Myocardial Ischemia Reperfusion in Rats Through Keap1/Nrf2 Pathway
Source: Front Mol Biosci. 2021 Apr 30;8:655619. doi: 10.3389/fmolb.2021.655619 (PMC8119885; doi:10.3389/fmolb.2021.655619)

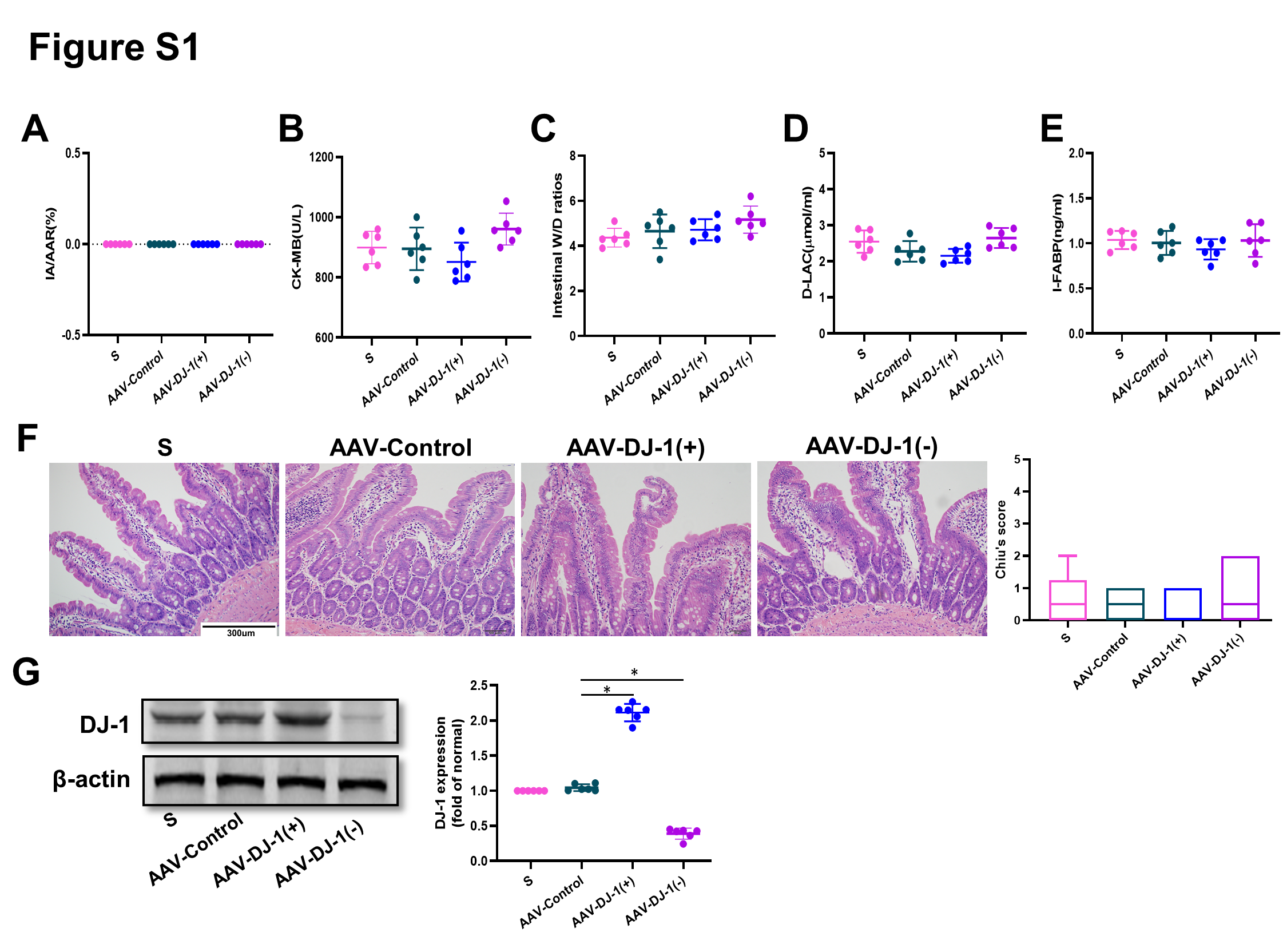

Supplement: Supplementary Figure 1 — Verify the safety of AAV. (A) Infarct area relative to the area at risk (IA/AAR × 100%). (B) serum CK-MB level. (C) Intestinal water W/D ratios. Serum concentrations of D-LA (D) and I-FABP (E). (F) Histopathologic changes of the small-intestinal mucosa under light microscopy imaging (H&E staining, Scale bar = 300 μm). Intestinal mucosa injury was graded by Chiu’s score. (G) Expression of DJ-1 level. S, Sham; IR, ischemia reperfusion; IPo, ischemic postconditioning. Data are mean ± SD or Box-Whisker’s plot (n = 6); ∗P < 0.05. [file Image_1.TIF]
